# Triacontanol regulates morphological traits and enzymatic activities of salinity affected hot pepper plants

**DOI:** 10.1038/s41598-022-06516-w

**Published:** 2022-03-08

**Authors:** Mubeen Sarwar, Sumreen Anjum, Muhammad Waqar Alam, Qurban Ali, C. M. Ayyub, Muhammad Saleem Haider, M. Irfan Ashraf, Wajid Mahboob

**Affiliations:** 1 Department of Horticulture, University of the Punjab, Lahore, Pakistan; 2 Institute of Botany, Univesity of the Punjab, Lahore, Pakistan; 3 Department of Plant Pathology, Univesity of Okara, Okara, Pakistan; 4 Institute of Molecular Biology and Biotechnology, The University of Lahore, Lahore, Pakistan; 5 Insitute of Horticultural Sciences, University of Agriculture, Faisalabad, Pakistan; 6 Department of Plant Pathology, University of the Punjab, Lahore, Pakistan; 7 Plant Physiology Division, Nuclear Institute of Agriculture, Tandojam Hyderabad, Pakistan

**Keywords:** Plant sciences, Plant stress responses

## Abstract

Potential role of triacontanol applied as a foliar treatment to ameliorate the adverse effects of salinity on hot pepper plants was evaluated. In this pot experiment, hot pepper plants under 75 mM NaCl stress environment were subjected to foliar application of 25, 50, and 75 µM triacontanol treatments; whereas, untreated plants were taken as control. Salt stress had a significant impact on morphological characteristics, photosynthetic pigments, gas exchange attributes, MDA content, antioxidants activities, electrolytes leakage, vitamin C, soluble protein, and proline contents. All triacontanol treatments significantly mitigated the adversative effects of salinity on hot pepper plants; however, foliar application triacontanol at 75 µM had considerably improved the growth of hot pepper plants in terms of plant height, shoot length, leaf area, plant fresh/dry biomasses by modulating above mentioned physio-biochemical traits. While, improvement in gas exchange properties, chlorophyll, carotenoid contents, increased proline contents coupled with higher SOD and CAT activities were observed in response to 75 µM triacontanol followed by 50 µM triacontanol treatment. MDA and H_2_O_2 _contents were decreased significantly in hot pepper plants sprayed with 75 µM triacontanol followed by 50 µM triacontanol foliar treatment. Meanwhile, root and shoot lengths were maximum in 50 µM triacontanol sprayed hot pepper plants along with enhanced APX activity on exposure to salt stress. In crux, exogenous application triacontanol treatments improved hot pepper performance under salinity, however,75 µM triacontanol treatment evidently was more effective in mitigating the lethal impact of saline stress via controlling the ROS generation and increment in antioxidant enzyme activities.

## Introduction

Salt stress is believed to have the most devastating implication among abiotic stresses, which causes loss in crop productivity around the world^1–3^ and adversely affects almost every aspect of the physiology and biochemistry of plants^4^ significantly reduces yield^5,6^. According to a report, almost 25–30% of total world agricultural land is saline that showed rapid degeneration of fertile land especially in arid and semi-arid regions^7,8^. Salinity impairs plant ionic homeostasis and water potential under high salt concentration and also disturbs the processes of photosynthesis and protein production^9–11^. Plant exposure to salt stress triggers the production of reactive oxygen species (ROS) which cause protein denaturation, DNA damages, chlorophyll degradation, and cell membrane permeability by promoting the lipid peroxidation status of plants^12,13^.

Hot pepper (*Capsicum annuum* L.) being an important vegetable crop is considered salt sensitive due to which its growth, productivity, and quality characteristics are severely hampered upon exposure to salt stress conditions^14–16^. Application of phytohormones play a vital role in plant growth and productivity by comMunicating several signals among as well as inside the cells; but their internal level endures substantial variation under saline stress^5,17,18^. It has been revealed that salinity stress caused a decline in the production of plant growth regulators^19^, thus, foliage application of these growth regulators can even enhance their endogenous levels under salinity^20,21^. Triacontanol is a potential plant growth promoter and its foliar application has been reported to influence physiological and biochemical processes in plants under saline conditions^13,22^. Previous instances have proved that foliar supplementation of triacontanols at various growth stages resulted in improved productivity of wheat, rice, and cucumber^4,23,24^. In another study, triacontanol enhanced plant growth by modifying many metabolic processes facilitating water uptake, cell division, chlorophyll synthesis, photorespiration, photosynthesis; thereby, boosting the activities of a few key enzymes mineral nutrient status^2,23,24^. Moreover, the application of triacontanol has also been reported to enhance enzymatic and non-enzymatic antioxidants production to mitigate the negative effect of salt stress^4,17,25^. Keeping in view these facts, it is evident that triacontanol has positive impacts on plant growth even under abiotic stresses like salinity stress, and its role in comMercially and nutritionally important vegetables like hot pepper is yet to be explored. Therefore, the current study was designed to investigate the potential role of foliarly applied triacontanol in alleviating salt stress and improving the growth of hot pepper plants under salinity-induced oxidative stress.

## Materials and methods

A pot experiment of hot pepper plants was conducted to evaluate the efficacy of foliar application of triacontanol under salt stress conditions. It has been confirmed that the experimental samples of plants, including the collection of plant material, complied with relevant institutional, national, and international guidelines and legislation with appropriate permissions from Institute authorities of Institute of Agricultural Sciences, University of the Punjab, Lahore Pakistan for collection of plant specimens. This study consisted of four levels (0, 25, 50, and 75 µM) of triacontanol spray on hot pepper plants under salinity stress (75 mM NaCl). The study was comprised of five treatments and four replications, each treatment consisted of 30 pots containing one plant of hot pepper. Seeds of hot pepper plants sterilized with sodium hypochlorite 0.1% solution followed by rinsing with distilled water were sown in 9L pots at the research area of Institute of Agricultural Sciences, University of the Punjab, Lahore. The day/night temperature was 35/30 °C with a photoperiod of 16/8 h light/dark and R.H 45%. Seedlings were watered with Hoagland solution according to the moisture status of the growing medium. Salt stress at 75 mM NaCl was induced in potted hot pepper plants after 50 days of emergence. Foliar spray of triacontanol was used twice, first spray after 72 h of stress imposition; while, 2^nd^ spray was applied at flowering stage. After one week of 2nd spray, a sample of hot pepper plants was harvested for morphological characteristics, fresh leaves were collected and instantly stored for biochemical analysis at − 80 °C.

### Growth attributes of hot pepper plants

Growth attributes of hot pepper shoot and root length were noted using meter rod; while, fresh weight was taken with digital balance and dry weight was calculated by drying hot pepper leaf samples in the oven for 72 h at 65 °C^2^. Leaf area was taken using leaf area meter (Model: CL-01, Hansatech Instrument, UK).

### Gaseous exchange attributes of hot pepper plants

Gaseous exchange characteristics such as photosynthetic rate (*Pn*), CO_2_ assimilation, intercellular CO_2_ concentration (*C*i), and transpiration rate (*E*) was measured using a portable infrared gas analyzer (Analytical Development Company, Hoddesdon, England) from the intact leaves of hot pepper plants^26,27^.

### SPAD value and photosynthetic pigments concentrations of hot pepper plants

SPAD value of the fully expanded hot pepper leaves were estimated with help of a portable chlorophyll meter (Konica Minolta Sensing, Inc; Japan) by following the method described by Sarwar et al.^2^. Chlorophyll a and b pigments were measured by extracting hot pepper plant samples in 80% acetone solution through mechanical grinding^28^. Optical densities of both chlorophyll pigments and carotenoids from prepared hot pepper sample solutions were measured at 663 nm, 645 nm, and 480 nm by using the following formulas:

$$Chl. a={0.0127D}_{663}- {0.00269D}_{645}$$

$$Chl. b= {0.0229D}_{645}- {0.00468D}_{663}$$

### Antioxidative enzyme activities and lipid peroxidation of hot pepper plants

Hot pepper leaves (500 mg) were homogenized in K_3_PO_4_ buffer solution (pH 7.0) added with 1 mM ethylene diamine tetra-acetic acid and 1% (w/v) soluble polyvinyl pyrrolidone (PVP). Prepared solutions were centrifuged at 20,000*g* to separate the supernatant that was further used for the determination of superoxide dismutase (SOD), catalase (CAT), peroxidase (POX), and ascorbate peroxidase (APX) activities.

Superoxide dismutase activity (SOD) was assessed by calculating its capability to hinder the photo-chemical decline of nitro-blue tetrazolium chloride (NBT)^29^; while Catalase (CAT) was measured by the procedure of Dhindsa et al.^30^ where one unit CAT was specified as a change in absorbance of 0.01 units per min. The POD activity was calculated by the described procedure of Plewa et al.^31^ and the activity of ascorbate peroxidase (APX) was analyzed by observing a reduction in ascorbic acid (ASA) with spectrophotometry^32^. Lipid peroxidation was determined by calculating malondialdehyde (MDA) from hot pepper leaves that were produced due to reaction with 2-thiobarbituric acid as scrutinized by Heath and Packer^33^. In short, supernatant that was prepared after a number of steps was taken and added to 20% TCA having 0.5% 2-thiobarbituric acid in 4 ml solution which was boiled for 30 min at 90 °C followed by centrifugation at 10,000×*g* and absorbance was detected at 532 and 600 nm.

### Electrolyte leakage, ascorbic acid contents, soluble protein and proline contents

Electrolyte leakage percentage was calculated from leaf samples of hot pepper plants introduced by Lutts et al.^34^. The ground tissue of hot pepper plants was added with 10% TCA and centrifuged to get supernatant for determination of ascorbic acid contents^35^. Soluble protein contents were measured using bovine serum albumin as a protein standard^36^. Proline contents of hot pepper plants samples were calculated by homogenized fresh leaf tissue (0.5 g) in 3% sulfosalicylic. Sample solutions prepared as by standard protocol were run at 520 nm and proline contents were determined from a standard curve^37^.

### Statistical analysis

Data for different parameters was analyzed in a factorial arrangement under complete randomized design (CRD) and results were interpreted using analysis of variance technique followed by LSD tests at a (0.05) significance level by using statistix 8.1.

## Results

### Growth attributes of hot pepper plants

It is evident from results that salt stress influenced plant growth and physio-biochemical attributes of hot pepper; while, maximum shoot length, root length, fresh/ dry biomass, and length were recorded in unstressed control plants. Plants subjected to 75 mM NaCl stress illustrated a decline in plant growth in terms of reduced shoot and root length, plant fresh/dry biomasses, and leaf area. Thus, exogenous application of triacontanol (25, 50, and 75 µM) significantly improved the salt tolerance of hot pepper plants and maintained better growth and biomass compared with plants in which foliar triacontanol was not applied. Among the triacontanol treatments, 75 µM triacontanol proved to be most effective followed by 50 µM and 25 to µM triacontanol in enhancing the shoot length and other growth parameters including plant fresh/dry biomasses and leaf area; whereas, root length was relatively longer in case of 50 µM triacontanol treatment (Table 1).

**Table 1:** Effect of triacontanol spray on morphological characters of hot pepper plants under salt stress.

	Treatments	Shoot length (cm)	Root length (cm)	Plant F.W. (g)	Plant D.W. (g)	Plant height (cm)	Leaf area (cm^2^)
T_0_	Control	37.46 ± 1.47a	13.77 ± 1.67a	58.77 ± 1.43a	11.02 ± 0.40 a	39.53 ± 1.23 a	233.24 ± 7.45 a
T_1_	75 mM NaCl (S)	22.30 ± 1.89d	7.55 ± 0.43c	37.55 ± 4.60c	4.30 ± 0.61c	25.66 ± 1.81 c	160.61 ± 10.26 b
T_2_	Triacontanol 25 µM + S	25.60 ± 1.23 cd	8.77 ± 0.87bc	41.10 ± 1.70 c	6.60 ± 0.57bc	29.28 ± 0.10 bc	165.50 ± 7.93 b
T_3_	Triacontanol 50 µM + S	28.57 ± 0.28bc	11.07 ± 0.80ab	43.82 ± 1.76c	7.57 ± 0.65bc	33.22 ± 2.56 ab	181.83 ± 10.98 b
T_4_	Triacontanol 75 µM + S	31.40 ± 1.10b	10.65 ± 0.96b	50.90 ± 1.45b	8.64 ± 0.30b	35.44 ± 4.02 ab	209.84 ± 4.68 a

Means sharing the same letter for a parameter, do not differ significantly at p ≤ 0.05; F.W. = Fresh Weight; D.W. Dry weight.

### Gaseous exchange attributes of hot pepper plants

Unstressed hot pepper plants exhibited maximum *Pn* (25.04 μmol CO2 H_2_O m^−2^ s^−1^), CO_2_ assimilation rate (0.82 µmol m^−2^ s^−1^), *C*_i _rate (358.68 µmol mol^−1^), and T*r* rate (9.17 *mM*ol H_2_O m^−2^ s^−1^) than 75 mM NaCl exposed plants; although, foliar application of all triacontanol concentrations alleviate the devastating effects of 75 mM NaCl salt stress exposure by maintaining better photosynthetic rates (*Pn*), CO_2_ assimilation rate as well as *C*_i_ rate concentration and transpiration rates (T*r*). Among triacontanol treatments, foliar application of 75 µM triacontanol proved to most suitable concentration by exhibiting a higher P*n* rate (21.02 μmol CO_2_ m^−2^ s^−1^), CO_2 _assimilation rate (0.71 µmol m^−2^ s^−1^) and T*r* rate (7.46 *mM*ol H_2_O m^−2^ s^−1^) followed by 50 µM triacontanol treatment (Table 2).

**Table 2:** Effects of triacontanol spray on gas exchange attributes of Hot pepper plants under salt stress.

	Treatments	Pn rate	CO_2_ rate	C_i_rate	Tr rate
T_0_	Control	25.04 ± 0.93	0.82 ± 0.05a	358.68 ± 0.73a	9.17 ± 1.01a
T_1_	75 mM NaCl (S)	16.22 ± 1.04b	0.47 ± 0.03b	323.72 ± 4.98a	6.06 ± 0.76b
T_2_	Triacontanol 25 µM + S	18.63 ± 0.78b	0.54 ± 0.02ab	347.04 ± 18.02a	7.13 ± 0.58ab
T_3_	Triacontanol 50 µM + S	19.24 ± 2.37b	0.64 ± 0.06ab	352.05 ± 12.23a	7.24 ± 0.74ab
T_4_	Triacontanol 75 µM + S	21.02 ± 2.42ab	0.71 ± 0.22ab	346.35 ± 19.61a	7.46 ± 0.65ab

Means sharing the same letter for a parameter, do not differ significantly at *p* ≤ 0.05; Pn = photosynthesis rate; Tr = transpiration rate; Ci = internal CO_2_.

### SPAD value and photosynthetic pigments concentrations of hot pepper plants

Results revealed that SPAD value, chlorophyll a, chlorophyll b, and carotenoid pigments of hot paper plants were degraded on exposure to salt stress; whereas, the highest SPAD value 28.52 and carotenoid contents (5.28 mg g^−1^ FW) were recorded in unstressed hot pepper plants followed by triacontanol treatments as a foliar application of triacontanol treatments had mitigated adverse effects of salinity. Likewise, chlorophyll a and chlorophyll b pigments were also high in unstressed hot pepper plants followed triacontanol sprayed plants, Results revealed that foliar application of triacontanol (75 µM) showed maximum leaf chlorophyll content and significantly retained the highest SPAD value (23.40 mg g^−1^ FW) as well as produced maximum chlorophyll a (15.14 mg g^−1^ FW), chlorophyll b (5.07 mg g^−1^ FW) and carotenoid contents (4.84 mg g^−1^ FW) (Table 3).

**Table 3:** Effects of triacontanol spray on photosynthetic pigments of Hot pepper plants under salt stress.

	Treatments	Chl. (SPAD)	Chl. *a*	Chl. *b*	Carotenids
T_0_	Control	28.52 ± 1.26 a	18.12 ± 1.49 a	6.71 ± 1.55 a	5.28 ± 0.51 a
T_1_	75 mM NaCl (S)	15.30 ± 0.84c	11.24 ± 0.90 c	3.71 ± 0.17 b	3.68 ± 0.23 b
T_2_	Triacontanol 25 µM + S	19.10 ± 0.47bc	13.79 ± 1.16 bc	4.15 ± 0.45 b	3.92 ± 0.75 ab
T_3_	Triacontanol 50 µM + S	20.32 ± 1.03bc	14.39 ± 1.31 bc	4.37 ± 0.37 ab	4.20 ± 0.47 ab
T_4_	Triacontanol 75 µM + S	23.40 ± 1.55b	15.14 ± 0.59 ab	5.07 ± 0.19 ab	4.84 ± 0.22 ab

Means sharing the same letter for a parameter, do not differ significantly at *p* ≤ 0.05; Chl = Chlorophyll.

### Antioxidative enzyme activities and lipid peroxidation of hot pepper plants

Moreover, hot paper plants showed amplification in enzymatic activities of antioxidants i.e. superoxide dismutase (SOD), catalase (CAT), peroxidase (POX), and ascorbate peroxidase (APX) under 75 mM NaCl salt stress; while, foliar application of triacontanol revealed further improvement in enzymatic activities. Maximum activity of SOD (121.4 Units mg^−1^ protein) and POX (11.4 Units mg^−1^ protein) were observed for 75 µM triacontanol; while a foliar spray of 50 µM triacontanol exhibited the highest values for CAT (0.177 Units mg^−1^ protein) and APX activities (0.193 µmol mg^−1^ protein min^−1^) (Table 4). On the other hand, H_2_O_2_ contents were increased under salinity; as H_2_O_2_ contents were at the lowest (19.36 μmol kg^−1^ FW) in unstressed hot pepper plants and increased to significantly higher with H_2_O_2 _contents up to 31.91 μmol kg^−1^ FW in 75 mM NaCl stressed hot pepper plants. Foliar application of triacontanol sprays resulted in a reduction of H_2_O_2_ contents as lowest concentration was recorded in 75 µM triacontanol sprayed hot pepper plants which were 25.07 µmol g^−1^ FW. In case of lipid peroxidation, maximum MDA content was produced in response to 75 mM NaCl treatment whereas foliar application of triacontanol had significantly reduced MDA production by showing lowest value for MDA content (38.02 µmol g^−1^ FW) in hot paper plants treated with 75 µM triacontanol followed by 50 µM triacontanol under salinity (Table 4).

**Table 4:** Effects of triacontanol spray on antioxidants activities and lipid peroxidation of Hot pepper plants under salt stress.

	Treatments	SOD	POX	CAT	APX	H_2_O_2_	MDA
T_0_	Control	52.02 ± 0.84e	2.52 ± 0.2d	0.062 ± 0.09c	0.126 ± 0.09c	19.36 ± 1.49 d	22.27 ± 1.02e
T_1_	75 mM NaCl (S)	73.55 ± 2.63d	5.30 ± 0.4c	0.082 ± 0.09b	0.142 ± 0.07bc	31.91 ± 2.50 a	187.03 ± 4.9a
T_2_	Triacontanol 25 µM + S	91.10 ± 2.44c	8.10 ± 0.4b	0.134 ± 0.09a	0.157 ± 0.029bc	30.36 ± 0.43 ab	103.28 ± 3.6b
T_3_	Triacontanol 50 µM + S	101.3 ± 2.19b	9.07 ± 0.5b	0.177 ± 0.07a	0.193 ± 0.003a	27.03 ± 1.49 bc	84.04 ± 2.48c
T_4_	Triacontanol 75 µM + S	121.4 ± 2.87a	11.40 ± 0.2a	0.157 ± 0.02a	0.178 ± 0.010ab	25.47 c	38.02 ± 4.11d

Means sharing the same letter for a parameter, do not differ significantly at *p* ≤ 0.05; SOD = Superoxide dismutase; POX = Peroxidase; CAT = catalase; APX = Ascorbate peroxidase, MDA = malondialdehyde.

### Electrolyte leakage, ascorbic acid contents, soluble protein and proline contents

Electrolyte leakage of unstressed hot pepper plants was 15.89% which was significantly low compared to electrolyte leakage of 75 mM NaCl stressed hot pepper plants which were 26.30%; however, foliar application of triacontanol increased membrane integrity by maintaining significantly reduced electrolyte leakage than unsprayed 75 mM stressed hot pepper plants. Among all triacontanol treatments, 75 µM concentration performed exceptionally better and maintained lowest electrolyte leakage (18.28%) followed by 50 µM and 25 µM treatments. Likewise, parallel observations were noticed for ascorbic acid contents, soluble protein and proline contents in unstressed, 75 NaCl stressed and foliarly triacontanol sprayed hot pepper plants. Unstressed hot pepper plants exhibited significantly higher ascorbic acid contents (60.29 mg 100 g^−1^) and soluble protein contents (42.60 mg g^−1^ FW) sprayed which were decreased to 38.80 and 26.58, respectively. Foliar application of triacontanol ameliorated the harmful effect of salt stress and triacontanol at 75 µM performed exceptionally better by exhibiting 52.94 mg 100 g^−1^ ascorbic acid contents which were significantly higher than all other triacontanol treatments; similarly, soluble protein contents were significantly higher in triacontanol sprayed hot pepper plants although 50 and 75 µM triacontanol treatments showed at values for soluble protein contents. Meanwhile, proline contents were increased significantly when hot pepper plants were exposed to 75 mM NaCl stress; although, hot pepper plants ameliorated the harmful effect of saline stress and maintained higher values of proline contents than control (Table 5).

**Table 5:** Effects of triacontanol spray on electrolyte leakage and biochemical attributes of Hot pepper plants under salt stress.

	Treatments	E.leakeage (%)	Vitamin C	Sol. Proteins	Proline
T_0_	Control	15.89 ± 0.76 d	60.29 ± 1.66 a	42.60 ± 2.92 a	24.37 ± 1.43 b
T_1_	75 mM NaCl (S)	26.30 ± 0.77 a	38.80 ± 3.84 c	26.58 ± 0.80 c	27.92 ± 1.20 ab
T_2_	Triacontanol 25 µM + S	23.30 ± 0.40 bc	41.02 ± 2.29 c	28.24 ± 1.55 bc	29.03 ± 1.48 a
T_3_	Triacontanol 50 µM + S	21.30 ± 1.80 bc	45.47 ± 2.06 bc	32.58 ± 2.01 b	30.18 ± 0.98 a
T_4_	Triacontanol 75 µM + S	18.28 ± 1.24 cb	52.94 ± 1.11 ab	33.02 ± 1.39 b	31.37 ± 0.50 a

Means sharing the same letter for a parameter, do not differ significantly at *p* ≤ 0.05; Sol. Protein = soluble protein.

## Dicussion

Hot pepper is regarded as a sensitive to moderately sensitive crop to salt stress^15,38^. Growing hot pepper under saline conditions severely affects the growth and productivity of plants^14,17^. A decline in the growth of hot pepper plants grown in pots under saline stress was confirmed by our findings in Table 1. A decrease in growth and production of hot pepper plants might be due to restricted water absorption, decreased metabolic activities as a result of sodium or chloride toxicity, and specific nutrient deficiency produced via ionic intrusion^1,2^. However, foliar feeding of plant growth regulators can reduce such lethal impacts of saline stress on plants^39,40^. Our results that foliar application of triacontanol significantly improved growth attributes of stressed plants have concurred with the findings of Singh et al.,^24^, where triacontanol treatment encouraged the growth of ginger plants under saline stress. It might be attributed to the synergetic role of triacontanol with gibberellic acid and cytokinins to regulate growth, metabolic processes, and yield of crops^41^. Moreover, triacontanol encouraged the development of second messenger 9-b- L(+) adenosine, which is similar to the cytokinins structure^42^ that could have facilitated an increase in leaf area and photosynthesis of hot pepper correlated with a shoot and root length as well as their fresh and dry biomasses^43^.

Gaseous exchange properties of hot pepper plants exposed to salt stress as presented in Table 2 were comparable with previous findings reported in different crops i.e. wheat and cucumber^2,23,45^. Impaired photosynthesis rate under salinity might be attributed to oxidative damage to imperative photosynthetic cells^2,46^ or decline in stomatal conductance that ultimately restricts the availability of carbon dioxide to leaf tissues, resulted from an antagonistic imbalance of Na^+^ ion on K^+^ which is required for stomatal activity^45^. Exogenously applied triacontanol had significantly induced salt tolerance in hot pepper by positively modulating gas exchange properties as stated in rice crop under saline grown under saline conditions^47,48^. This improvement in gas exchange attributes by triacontanol, proved its well-established role in stomata regulation by up-regulating photosynthetic genes^49^, increasing CO_2_ exchange rate^23^; and enhanced rubisco activity which ultimately boosts photosynthesis^50^. Triacontanol treatments resulted in a rapid increase in activities of a specific secondary messenger like 9-b-L(−) adenosine, which could lead towards quick physiological responses^51^. Progressive impacts of triacontanol on photosynthesis rate may be due to improvement in the efficiency of photosystem II under saline environment and revealed that triacontanol improves stress tolerance in hot pepper by stabilizing photosynthetic pigments^52^.

Chlorophyll contents of hot pepper plants subjected to salt stress were significantly degraded as presented in Table 3; and a similar decline in chlorophyll content has previously been reported in hot pepper crops^1,53^. Salinity stress-induced accumulation of toxic ions and physiological water deficit in leaves delayed the chlorophyll biosynthesis and also accelerated the degradation of original chlorophyll^54^. However, exogenous application of triacontanol had a positive impact on chlorophyll pigments integrity, as in our results 75 µM triacontanol proved most effective in retaining the highest SPAD value as well as chlorophyll a, b, and carotenoid contents (Table 3). The improved chlorophyll content due to foliar exposure to triacontanol is presumed to be associated with stability membrane strength, which remains intact in response to triacontanol under saline conditions.

Enzymatic activities of SOD, CAT, POX, and APX were amplified hot pepper plants in response to salinity as well as foliar spray of triacontanol over the controls (Table 4); as, antioxidants are believed to have a key role in improving salt tolerance in plants^55^. The rise in antioxidant activities plays a vital role in the detoxification of ROS which leads toward the establishment of a balance between production and scavenging of ROS and prevents hot pepper plants from adverse effects of salinity. An increment in antioxidant enzyme activity under saline stress was also reported in tomato^12^ and maize crop^1,2,23,56^. In this study, triacontanol-induced improvement in growth might be attributed to its influence on the actions of antioxidant enzymes, i.e., SOD, CAT, POX, and APX under salinity stress^23,57^. Our results verified that salinity-induced oxidative stress produced H_2_O_2_ content and modulated lipid peroxidation in terms of enhanced malondialdehyde (MDA) content in hot pepper under saline condition (Table 4); as increment in MDA content was also reported by Ozdemir et al.^53^ in hot pepper. Application of triacontanol reduced lipid peroxidation and H_2_O_2_ production significantly under saline stress compared to non-sprayed plants and similar observations were reported by Verma et al.^58^ where triacontanol decreased MDA in peanut crops. Triacontanol hampered MDA and H_2_O_2_ production could be related to increased antioxidant activity or enhanced antioxidant production as observed in opium poppy by Khan et al.^17^.

Imposition of salt stress to hot pepper destabilized membrane integrity and resulted in increased electrolyte leakage content as shown in hot pepper plants (Table 5), higher electrolyte leakage in salinity-induced hot pepper plants could be due to the production of reactive oxygen species that in turn might have caused oxidation of phospholipids molecules in the cell membrane. Triacontanol foliar application reduced electrolyte leakage due to enhanced water uptake, augmented cell division and membrane stability by reducing oxidative stress^2,59^. Triacontanol plays an effective role in upregulating multiple physiological and biochemical pathways in plants^49^. An increment in proline contents was observed in the current study; as it is well known that endogenous level of free proline increases under saline conditions^54^; whereas, the concentration of soluble proteins and ascorbic acid contents of hot pepper plants were reduced upon induction of salt stress^60^. Proline has been reported to induce salt tolerance due to its role in osmotic adjustment and stabilizing the structure of organelles and macromolecules^61^. Our results illustrated that hot pepper plants treated with exogenous triacontanol showed improved leaf proline contents as well as soluble protein contents; these findings are in agreement with the observations recorded in green gram and sweet basil crops grown under saline environment^1,2,62,63^.

## Conclusions

Salt stress exhibited significantly reduced plant growth and development in unsprayed hot pepper plants. All concentrations of foliar triacontanol supplement were proved beneficial for stress alleviation in hot pepper plant; however, triacontanol at 75 µM was more beneficial as it significantly improved hot pepper quality attributes like plant fresh and dry biomasses, gaseous exchange properties, activities of antioxidant enzymes, cell membrane integrity, proline, ascorbic acid, and soluble protein. Hence, it was concluded that 75 µM was the most beneficial triacontanol treatment to alleviate 75 mM NaCl stress in pot-grown hot pepper plants.
